# Growth and Investigation of Annealing Effects on Ternary Cd_1−x_Mg_x_O Nanocomposit Thin Films

**DOI:** 10.3390/ma14164538

**Published:** 2021-08-12

**Authors:** Khalid Bashir, Nasir Mehboob, Muhammad Ashraf, Abid Zaman, Fozia Sultana, Abdul Faheem Khan, Asad Ali, Muhammad Mushtaq, Sarir Uddin, Khaled Althubeiti

**Affiliations:** 1 Department of Physics, Riphah International University, Islamabad 44000, Pakistan; nasir.mehboob@riphah.edu.pk (N.M.); kasadiiui@gmail.com (A.A.); 2 Optics Laboratories, P.O. Box 1021, Islamabad 44000, Pakistan; sm_ashr@yahoo.com; 3 Department of Chemistry, University of Science and Technology China, Hefei 230026, China; fsultana@mail.ustc.edu.cn; 4 Department of Materials Science & Engineering, Institute of Space Technology, Islamabad 44000, Pakistan; afkhan_ist@yahoo.com; 5 Faculty of Materials and Manufacturing, Beijing University of Technology, Beijing 100124, China; mushtaqphy009@yahoo.com; 6 Department of Physics, Government College Hayatabad, Peshawar 25000, Pakistan; sariruddin@uop.edu.pk; 7 Department of Chemistry, College of Science, Taif University, P.O. Box 11099, Taif 21944, Saudi Arabia; k.althubeiti@tu.edu.sa

**Keywords:** Cd_1−x_Mg_x_O films, blue shift, optoelectronics, band gap tailored, refractive index, oxide semiconductor

## Abstract

Thin films of Cd_1−x_Mg_x_O (CdMgO) (0 ≤ x ≤ 1) were investigated by depositing the films on glass substrates using the co-evaporation technique. The structural, surface morphological, optical, and electrical characteristics of these films were studied as a function of Mg content after annealing at 350 °C. The XRD analysis showed that the deposited films had an amorphous nature. The grain size of the films reduced as the Mg concentration increased, as evidenced by the surface morphology, and EDAX supported the existence of Mg content. It was observed that as the films were annealed, the transmittance of the CdMgO films saw an increase of up to 85%. The blue shift of the absorption edge was observed by the increase of Mg content, which was useful for enhancing the efficiency of solar cells. The optical band gap increased from 2.45 to 6.02 eV as the Mg content increased. With increased Mg content, the refractive index reduced from 2.49 to 1.735, and electrical resistivity increased from 535 Ω cm to 1.57 × 10^6^ Ω cm.

## 1. Introduction

Over the last two decades, the electronic industry has progressed drastically and, for materials scientists, has reached new horizons in the field of optoelectronics. The group II-VI elements have special importance for optoelectronics and chalcogenide semiconductors of this group such as Cd, Mg and Zn; their ternary alloys; and their oxides [1,2,3,4] are studied widely due to their excessive use in solar cells, photodetectors, photodiodes, gas sensors, transparent electrodes, and photo transistors [5,6,7,8,9,10], etc. There are few reports which regard CdO and MgO as a CdMgO ternary alloy. The compound CdO is a conducting material with a direct band gap of ~2.5 eV [11]. It shows transparent behavior in the visible region of electromagnetic spectrum. It has a cubic structure similar to rock salt, randomly oriented along the (111) plane and has a lattice constant of 4.70 Å. The compound CdO is a highly electrically conductive material owing to its increased concentration (~10^21^ cm^−3^) of free charge carriers. On the other hand, MgO has a rock-salt-like structure with a high band gap of 7.8 eV [12]. Its lattice constant is a = 4.24 Å, which is 1.11 times less than CdO. The compound MgO is a chemically and thermally stable oxide with a variety of applications in material science and technology. It also acts as a buffer layer for several materials such as metals, semiconductors and in many optoelectronics devices. However, CdO is not an efficient material for photovoltaics and optoelectronics due to its low optical band gap. It is reported that there are several dopants including indium, fluorine, lead, zinc, copper and gadolinium to improve the optical band gap of CdO [13,14,15,16,17,18]. Hence, by using MgO, which has a direct band gap of 7.8 eV with CdO, the optical band gap and UV luminescence may be modulated. The ions of Cd^++^ can be replaced with Mg^++^ ions to improve the electrical and optical characteristics of CdMgO. Consequently, the ternary combination CdMgO, which has a wide tunable band gap range of 2.5 to 7.8 eV, can be considered as a suitable compound for a buffer layer in solar cells and other optoelectronic devices. Despite their ability to tune the energy band gap, CdMgO films have received little attention. Only a few studies on the structural, morphological, electrical, and optical aspects of CdMgO thin films have been reported.

Different techniques have been used to fabricate CdMgO thin films such as radio frequency magnetron sputtering [19], the spray pyrolysis technique [20], metal organic chemical vapour deposition [21,22], and plasma-assisted molecular beam epitaxy [23]. However, these techniques are fairly expensive. As a result, a cost-effective and very simple approach for fabricating these films with improved properties is required.

In this work, the deposition of CdMgO (0 ≤ x ≤ 1) thin films is performed using the co-evaporation technique. To best of our knowledge, work on CdMgO thin films in the full range of composition by using the co-evaporation technique has not yet been reported. Furthermore, only a limited number of appropriate materials are accessible for the design of optical coatings. As a result, optical coating designers are constantly searching for new materials which are environmentally friendly and cost-effective, and have the appropriate refractive indices and minimal optical losses. The compond of CdMgO is a potential material that could be used in optical coatings and optoelectronic devices.

## 2. Experimental Procedure

The allot thin films of Cd_1−x_Mg_x_O (0 ≤ x ≤ 1) were fabricated by co-evaporating CdO and MgO materials while regulating the rate of evaporation in a variety of molar concentrations (x = 0.00, 0.25, 0.50, 0.75, 1.00). The MgO material was palletized into 2.5 mm wide discs with a 15 mm diameter using a hydraulic press. For almost 10 h, these discs were sintered at 1000 °C. The requisite thin films were deposited on soda lime glass substrates. To eliminate contaminants, these substrates were immersed in a concentrated H_2_SO_4_ solution for 12 h. These glass substrates were cleaned in an ultrasonic cleaner using acetone and isopropyl alcohol (IPA) after being thoroughly cleaned with detergent and washed in running water.

### 2.1. Sample Preparation

The films were deposited using an Edward E610A high vacuum coating equipment. The compound CdO was evaporated in a molybdenum boat, whereas MgO was evaporated in a tungsten boat using an electron beam. The chamber was evacuated to a pressure of (~1.1 × 10^−5^ mbar). The partial pressure of oxygen (~2.3 × 10^−4^ mbar) was fixed during the course of deposition. All of the films were made at ambient temperature (25 °C). Before deposition the chamber was heated up to 200 °C for degassing from the substrate surface. After cooling glow discharge to eliminate contaminants from the substrates, the chamber was brought to normal temperature. The substrates were revolved at 15 rpm to ensure homogeneous deposition. The film thickness and growth rate were monitored using a quartz crystal monitor FTM 7. The average thicknesses of the films were in the range 500–600 nm. The transmittance of the films was measured using Perkin Elmer Lambda 19 UV/VIS/NIR spectrophotometer and the software UV Win Lab. The transmittance data band gap and refractive indices of all these films were evaluated after they were fitted.

### 2.2. Characterization Techniques

The structural study of the aforementioned films was carried out at room temperature using a Bruker D8 discover diffractometer coupled with Cu Kα radiation in the scanning mode with 2θ ranging from 20 to 80°. Field emission scanning electron microscope (FE-SEM) TESCANMAIA3 with an Octane Elite EDAX detector was used to investigate the morphological aspects of the films. A high vacuum with suitably maintained acceleration voltages was used to investigate the surface of samples. The Energy dispersive X-ray Spectroscopy (EDAX) investigation for compositional analysis was executed at 20 kV voltage. The electrical resistivity of films was measured with a Keithly 2410-C, 1100 source meter using a two-probe approach.

## 3. Results and Discussion

**Figure 1:**
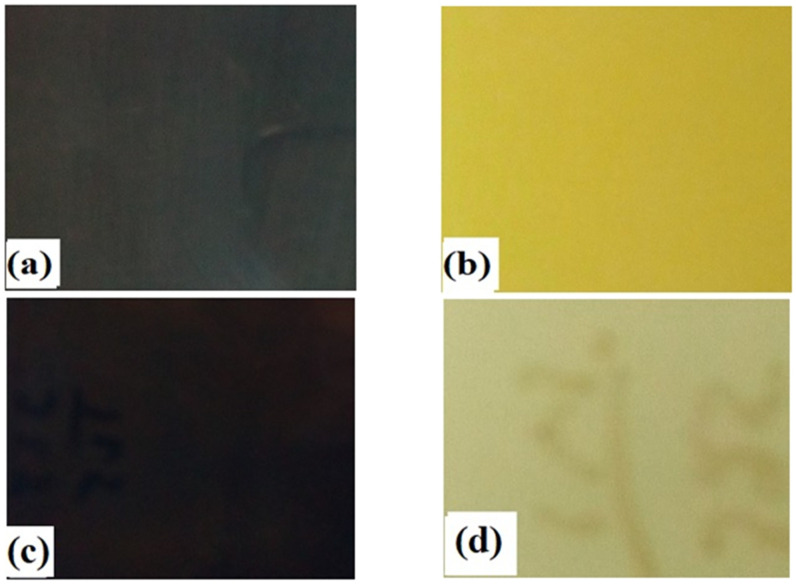
Image of thin films: (**a**) CdO before annealing; (**b**) CdO after annealing; (**c**) Cd_0.75_Mg_0.25_O before annealing; (**d**) Cd_0.75_Mg_0.25_O after annealing.

The thin films of CdO, MgO and CdMgO deposited at room temperature were found to be physically stable. No peel off, blisters or pinholes were present on the surface of the thin films. Furthermore, a simple tape test was performed on the films to check the adhesion of the films with the glass substrate and found that the films were fully intact with substrate. The deposition parameters had a strong influence on the adhesion of the films with the substrate, along with its quality and cleaning. The parameters were optimized after many trials to achieve a successful adhesion of the films with the substrates. The as-deposited films were annealed at 350 °C (optimized temperature) for three hours in air. After annealing, the transparency of the films improved significantly, as shown in Figure 1, and the color of the films changed from dark brownish to bright yellowish.

### 3.1. Structural Properties

**Figure 2:**
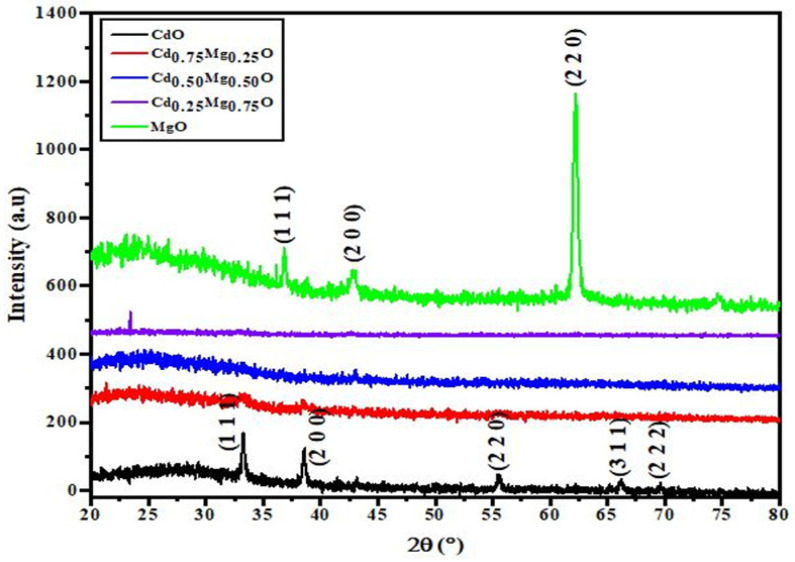
XRD profile of CdO, MgO and CdMgO.

The as-deposited CdMgO films were found to be amorphous in nature. The amorphous nature of the CdMgO films was attributed to the low-energy growth process, which was carried out at 25 °C. At low substrate temperature, the mobility of atomic species in thin films is insufficient to form an ordered structure. After annealing at 350 °C, the CdO and MgO films were converted to a polycrystalline nature where as CdMgO films again showed amorphous behavior (Figure 2). The amorphous behavior of CdMgO, even after annealing, was attributed to stresses in the films due to the radii difference between Cd and Mg ions.

The lattice constants, diffraction angles of major peaks and crystallite sizes of CdO and MgO thin films are summarized in Table 1.

### 3.2. Surface Morphology

**Figure 3:**
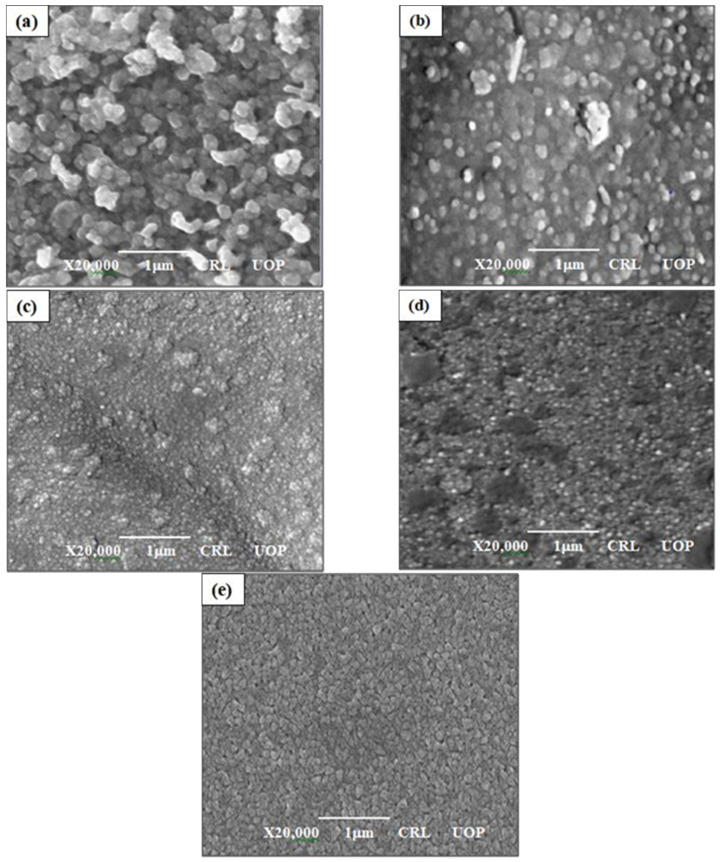
SEM images: (**a**) CdO; (**b**) Cd_0.75_Mg_0.25_O; (**c**) Cd_0.5_Mg_0.5_O; (**d**) Cd_0.25_Mg_0.75_O; (**e**) MgO annealed thin films.

Figure 3 shows the surface morphology of the annealed thin films. Figure 3a shows the surface morphology of CdO (undoped) films with interconnected spherical grains and some spaces. Figure 3b–e displays the influence of Mg concentration deposited on the CdO films; the grains started to modify into small nanocrystals/nanostructures throughout the whole surface of CdMgO. With the further increase of Mg content, images revealed that films became denser and more compact, which is required in photovoltaic applications to increase their efficiency.

**Figure 4:**
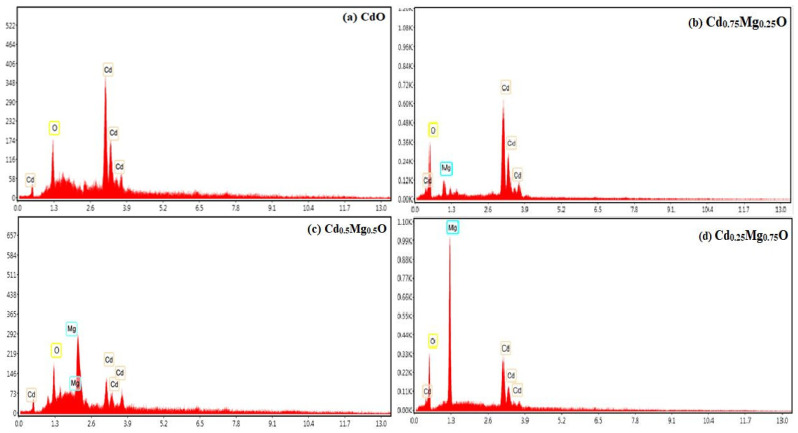
EDX spectra of (**a**) CdO; (**b**)Cd_0.75_Mg_0.25_O; (**c**) Cd_0.5_Mg_0.5_O; (**d**) Cd_0.25_Mg_0.75_O.

The amount of Mg deposited on the CdMgO thin films is revealed by EDX as shown in Figure 4a–d. With the increase in MgO evaporation, the concentration of Mg content in the films increased. There are some peaks of silicon which may have appeared due to the glass substrate. The atomic percentage composition of elements is shown in Table 2.

### 3.3. Optical Properties

The following equation [24], was used to elaborate the optical parameters of CdMgO thin films.

(1) $$T=\frac{Ax}{B-Cx\cos\phi +Dx^{2}},\;$$

- For such a type of semiconductor thin film, “*T*” represents the system’s typical transmittance [24]. The various variables are specified as follows: *x* = *exp*(−*αd*), ϕ = 4π*nd*/λ, *A* = 16 *n*^2^*s*, *B* = (*n* + 1)^3^(*n* + *s*^2^), *C* = 2(*n*^2^ − 1)(*n*^2^ − *s*^2^), *D* = (*n* − 1)^3^ (*n* − *s*^2^). Here
- *n* = the film refractive index
- *s* = the refractive index of the glass substrate
- *d* = film thickness
- *α* = the film’s absorption coefficient

The empirical formula for *n* is provided as [24,25]:

(2) $$n=a+\frac{b}{\lambda^{2}}\;(a,\;b\;\mathrm{are}\;\mathrm{constants})$$

The formula for *α* dependence of λ can be given as:

(3) $$\alpha =c+\frac{f}{\lambda }+\frac{g}{\lambda^{2}}\;(c,\;f\;\mathrm{and}\;g\;\mathrm{are}\;\mathrm{constants})$$

**Figure 5:**
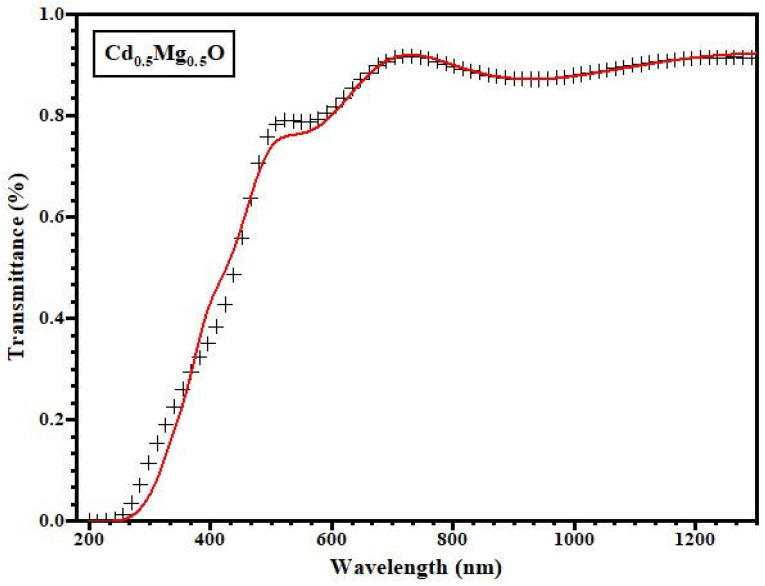
Transmittance of Cd_0.5_Mg_0.5_O thin film along with fitted curve.

The fit of Equation (1) to the transmittance plot of a representative Cd_0.5_Mg_0.5_O film is shown in Figure 5, revealing that Equation (1) offers a satisfactory correlation with the curve. In the high absorption zone, α was computed using the values of n and d determined from the fitted curve. The precise solution of Equation (1) for *x* in this scenario is:

(4) $$x=\frac{[C\cos(\phi )+A/T]-{\left[{\left(C\cos(\phi )+A/T\right)}^{2}-4BD\right]}^{1/2}}{2D}\;\alpha =-\frac{1}{d}\ln(x)$$

All of these parameters are defined above.

**Figure 6:**
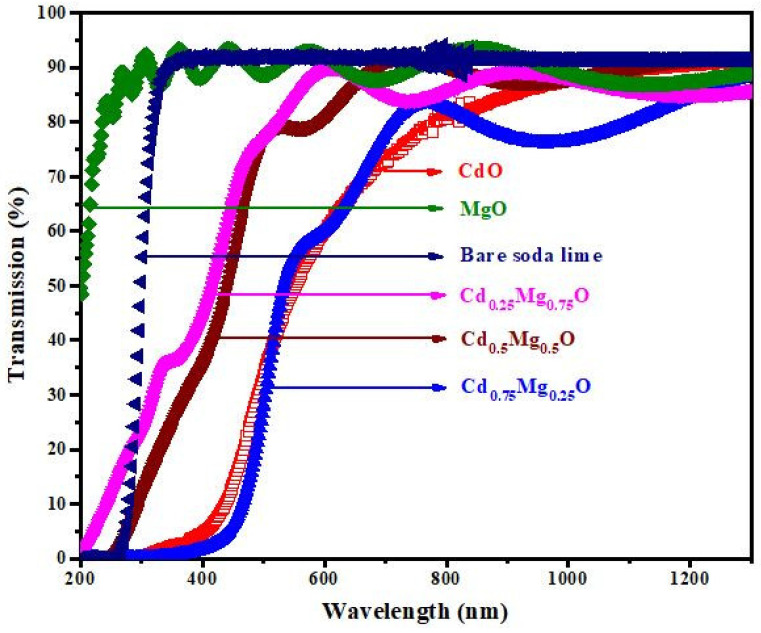
Transmittance spectra of CdO; Cd_0.75_Mg_0.25_O; Cd_0.5_Mg_0.5_O; Cd_0.25_Mg_0.75_O; and MgO annealed thin films.

Figure 6 depicts the fluctuation in transmittance data of CdMgO thin films from 200 to 1300 nm in the ultraviolet, visible and infrared regions. The spectra shown in Figure 5 and Figure 6 are plotted after subtracting glass transmittance values from the thin film’s transmittance.

The transmittance spectra of the films revealed that interference fringes lied in each spectrum. Two epilayers lied in the spectra: air-related and substrate-related epilayers. Interference between these interfaces took place and fringes appeared in the transmission spectra. When the Mg content of the films increased after annealing, the transmittance of the films increased by more than 80%. With the increase in Mg content, the absorption edge shifted toward a shorter wavelength. The Mg quantity in the samples caused structural changes, which ultimately shifted the absorption edge and band gap.

The direct energy band gap, *E_g_*, was calculated by using the well-recognized Tauc relation [25] $\alpha h\nu ={\left(h\nu -E_{g}\right)}^{1/2}$, where *hν* represents the photon energy. The energy band gap of CdMgO was estimated by extrapolating the linear portion of (*αhν*)*^2^* vs. (*hν*) curves to (*αhν*)^2^ = 0.

**Figure 7:**
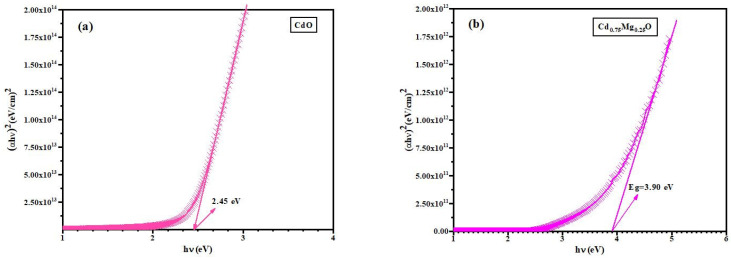

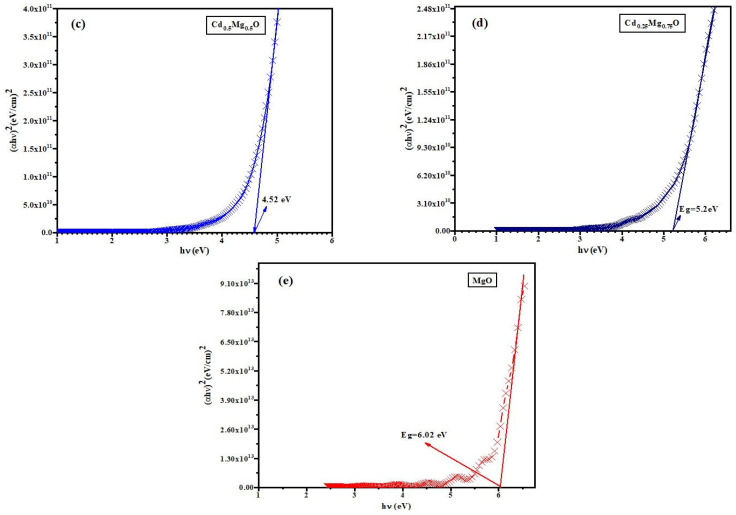
Energy band gap of (**a**) CdO (**b**) Cd_0.75_Mg_0.25_O (**c**) Cd_0.5_Mg_0.5_O (**d**) Cd_0.25_Mg_0.75_O (**e**) MgO annealed thin films.

The energy band gap of CdMgO increased from 2.45 to 6.02 eV when the Mg concentration increased, as seen in Figure 7a–e.

**Figure 8:**
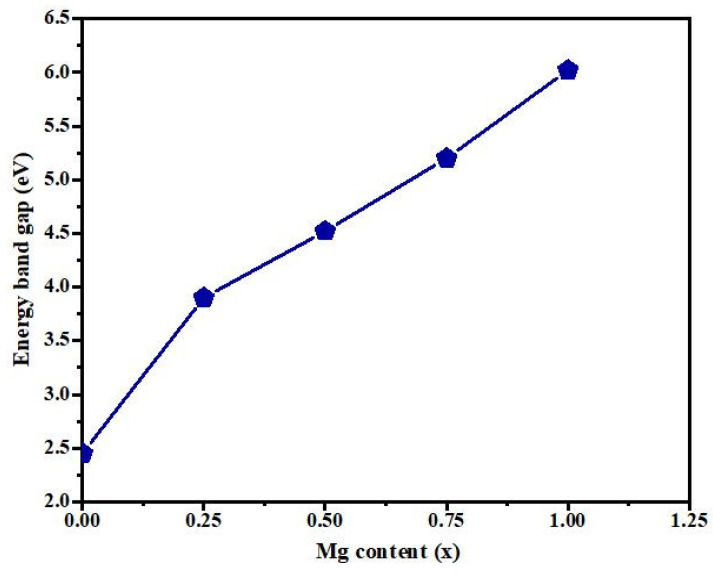
Variation of energy band gap with the increase in Mg content.

Figure 8 demonstrates how the energy band gap changes as the Mg content rises. The broadening of the band gap was associated with the smaller radii of Mg ions as compared with Cd ions. The size of the lattice constants reduced as the Mg content in CdMgO films increased, and the interaction between the wave functions associated with the valence electrons increased, resulting in a broadening of the energy band gap [26]. The refractive index of the films decreased from 2.49 to 1.74 with the increase of Mg content. This could have been related to a change in grain arrangement and an increase in strain in the film.

### 3.4. Electrical Properties

**Figure 9:**
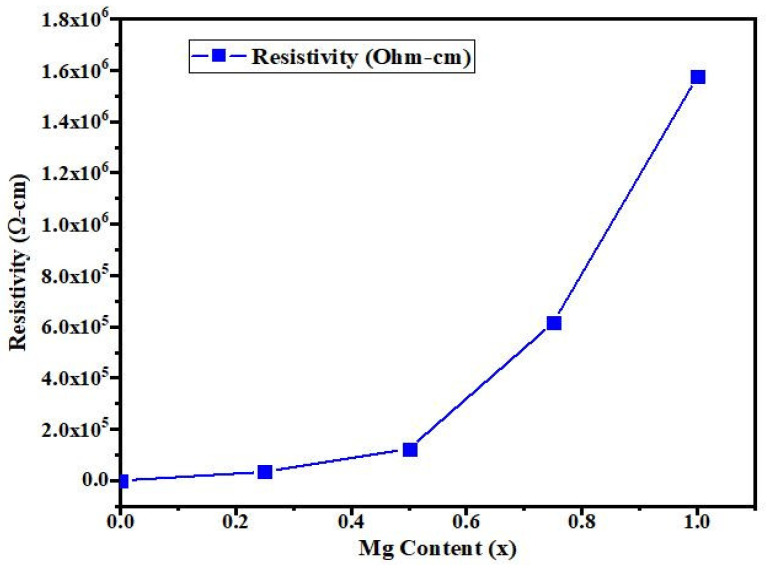
Electrical resistivity of CdMgO thin films with increasing Mg content.

Figure 9 shows the electrical resistivity of CdMgO thin films with increasing Mg content; the electrical resistivity of CdMgO proportionally to Mg content, from 535 Ω cm for pure CdO to 1.57 × 10^6^ Ω cm for CdMgO. This rise in resistivity was due to the increase in the band gap energies which occurred with the increase in Mg content in the films. The other factors for the increase in resistivity were due to the presence of oxygen vacancies, the reduction in the Cd interstitial site in the films and the increase in grain boundaries.

## 4. Conclusions

A detailed examination was conducted into the production of CdMgO thin films, utilizing the co-evaporation technique on soda lime glass substrates with a wide range of Mg content. It was investigated how magnesium content affected structural, morphological, optical, and electrical properties. X-ray diffraction showed that using the co-evaporation technique revealed that the thin films had amorphous behavior. SEM scans revealed that when the Mg content of the films increased, the surface morphology of the films improved, as did the transparency of the films. The energy band gap increased from 2.45 to 6.02 eV. With increased Mg concentration, the films’ refractive index reduced and their electrical resistance increased. The fabricated thin films of CdMgO could be a promising material used to create new optoelectronic devices, new coating materials, and for implementing in solar cell applications, based on the results of this study.

## Figures and Tables

**Table 1:** Lattice parameters and crystallite size of CdO and MgO.

Coating ID	Theta	Lattice Parameter d (Ǻ)	a (Ǻ)	Crystallite Size D (nm)
CdO	16.9	2.6498	4.5896	33.28
MgO	18.5	2.4277	4.2048	14.42

**Table 2:** Elemental composition of vacuum-annealed CdO, Cd_1−x_Mg_x_O, and MgO films (EDAX).

Element	Atom % (CdO)	Atom % (Cd_0.75_Mg_0.25_O)	Atom % (Cd_0.5_Mg_0.5_O)	Atom % (Cd_0.25_Mg_0.75_O)	Atom % (MgO)
Cadmium	52.60	39.70	26.91	13.31	-
Magnesium	-	12.10	25.21	37.18	53.80
Oxygen	47.40	48.20	47.88	49.51	46.20
Total	100	100	100	100	100
